# Seroprevalence of Q fever in cattle, sheep and goats in the Volta region of Ghana

**DOI:** 10.1002/vms3.160

**Published:** 2019-03-11

**Authors:** Sherry A. M. Johnson, John B. Kaneene, Kweku Asare‐Dompreh, William Tasiame, Ivy G. Mensah, Kofi Afakye, Shirley V. Simpson, Kwasi Addo

**Affiliations:** ^1^ School of Veterinary Medicine CBAS University of Ghana Legon Ghana; ^2^ Center for Comparative Epidemiology College of Veterinary Medicine Michigan State University East Lansing Michigan USA; ^3^ School of Veterinary Medicine Kwame Nkrumah University of Science and Technology Kumasi Ghana; ^4^ Noguchi Memorial Institute for Medical Research University of Ghana Legon Ghana

**Keywords:** Q fever, *Coxiella burnetii*, Ghana, livestock, zoonosis

## Abstract

Q fever is a zoonotic disease caused by *Coxiella burnetii,* a causative agent of abortion in livestock and febrile illness in humans. Outbreaks of human cases of Q fever have been reported in Australia and the Netherlands, which was linked to abortions in goat and sheep farms. In Ghana, information on Q fever in both livestock and humans is scanty. This study sought to determine the seroprevalence of Q fever in livestock in the Tongu area of the Volta region of Ghana. It was a cross sectional study with blood sampled from 204 cattle, 158 sheep and 100 goats. An indirect ELISA test was performed to detect Q fever antibodies in the serum of livestock. A total of 20 farms were sampled across the municipalities and an overall prevalence of Q fever was 21.6%. Specie‐specific prevalence was 28.4% (45/158) for sheep, 21.7% (45/204) for cattle and 10% (10/100) for goats. Abortions were reported on all the farms sampled and most farmers lived in close proximity to the farms sampled. Q fever is prevalent in the North Tongu area and requires the attention of the veterinary and health authorities, using the One‐ Health approach in order to control its occurrence and save lives.

## Introduction

Query fever (Q fever) is a zoonotic bacterial infection caused by *Coxiella burnetii (C. burnetii),* which causes abortion in livestock and, acute and chronic illness in humans. Cattle, sheep and goat are considered the main reservoirs of the disease, although the infection has been identified in dogs, cats, wildlife, reptiles and birds (Das *et al*. 2013; OIE, 2013). Q fever, previously considered a rare and regionally restricted disease (Eldin *et al*. 2017), has recently been shown to be globally spread, particularly in the tropics (Angelakis & Raoult 2010; Epelboin *et al*. 2016; Eldin *et al*. 2017), except in New Zealand (OIE, 2013). The causative agent of Q fever, *Coxiella burnetii* is considered a Category B agent of bioterrorism by the Centre for Disease Control (CDC) due to its route of transmission, low infective dose, high stability in the environment and prior weaponization (Kersh *et al*. 2013; Eldin *et al*. 2017).

Infections caused by *C. burnetii* usually present asymptomatically in livestock although the disease has been implicated in abortion, stillbirths, endometritis, mastitis and infertility (Radolakis *et al*. 2007; Angelakis & Raoult 2010; OIE, 2013). Infected animals shed *C. burnetii* in urine, faeces, milk, vaginal fluids, semen, placental and birth fluids (Guatteo *et al*. 2011; Rad *et al.,* 2014). In Africa, the highest seropositivity rates were reported from areas with the highest density of livestock (>100 per 100 inhabitants) and these included Mali, Burkina Faso, Nigeria and Central African Republic (Tissot‐Dupont *et al*., 2004).

Humans become infected with *C. burnetii* through the inhalation of aerosolized bacteria (Ratmanov *et al*. 2013) and consumption of contaminated unpasteurized milk. An outbreak of human cases of Q fever reported in the Netherlands was linked to abortions in dairy goat and sheep farms (Angelakis & Raoult 2010). Clinical cases of Q fever have been reported among the military and paramilitary deployed to Iraq (White *et al*. 2013). Clinical signs in humans include fever, fatigue, weight loss, pneumonia and hepatitis. Patients with underlying cardiac valve defects who get exposed to *C. burnetii* develop endocarditis or vascular infections (Wielders *et al*. 2015). Miscarriage and abortions have been reported as well (de Lange *et al*. 2015).

Q fever is considered a reportable disease in many countries including the United States of America (USA) and although the infection has been reported in all three countries bordering Ghana, namely, Cote d'Ivoire (Kanouté *et al*. 2017), Togo (Dean *et al*. 2013) and Burkina Faso (Ki‐Zerbo *et al*. 2000), Q fever is not part of the priority list of diseases under surveillance by the Ministry of Health (IDSR, 2002) or Veterinary Services Directorate (VSD, 2012). This implies that Q fever could be missed in differential diagnoses to be considered in abortion and infertility cases in livestock and in flu‐like and febrile conditions in humans. The only two publications on Q fever in Ghana to date have been in a cattle herd (Adu‐Addai *et al*. 2012) and children (Kobbe *et al*. 2008) in two separate regions. Given the mode of transmission and risk of exposure of Q fever to humans, a neglect of the disease among livestock farming communities could endanger the lives of those who work and live in close proximity to the livestock farms. The objective of this study was to determine the seroprevalence of Q fever in livestock in the Tongu area of the Volta region of Ghana.

## Materials and methodology

### Study area and design

**Figure 1:**
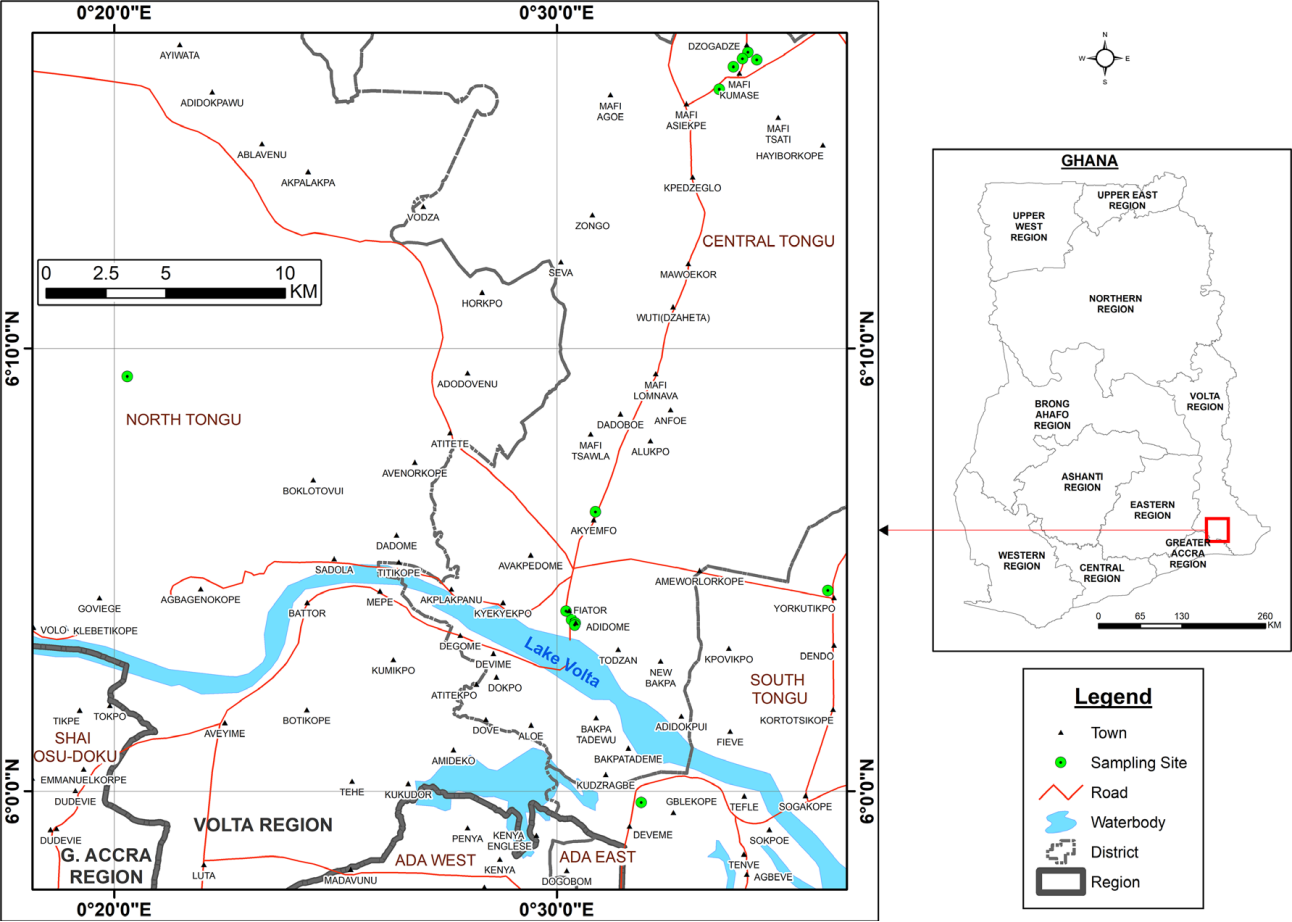
Q fever sampling sites in the Volta region of Ghana.

This was a cross sectional study in the Tongu districts made up of the North, Central and South Tongu of the Volta region of Ghana (Fig. 1). The site was chosen because of it being one of the regions with high livestock population density according the Veterinary Services Directorate. There are 25 districts in the Volta region with a total population of 61,904 cattle, 422,292 sheep and 617,165 goats (VSD annual report, 2016). The Tongu area is one of the largest livestock producing areas in Ghana with an estimated population of 34 564 cattle, 53 260 sheep and 78 310 goats (VSD 2012; Tasiame *et al*. 2016).

### Sample size

In the absence of a well‐determined prevalence of Q fever in livestock in Ghana, this study used published prevalence in livestock in Togo (Dean *et al*. 2013) as a proxy for calculating the sample size. This was mainly due to similarities in climatic and husbandry conditions in Ghana and Togo. Additionally, both countries do not apply *C. burnetii* vaccination to cattle, sheep and goats. Dean *et al*. (2013) reported a prevalence of *C. burnetii* infection of 14.8% in cattle, 14.4% in sheep, 8.3% in goat in Togo. As a result, minimum sample sizes of 191 cattle, 196 sheep and 95 goats were obtained. These were rounded up to 200 cattle, 200 sheep and 100 goats. This was also based on a 95% confidence level and a maximum allowable error of 5%.

### Sampling method/procedure

#### Selection of farms and animals

A list of cattle, sheep and goat farms in the region were obtained from the veterinary office in Adidome, Central Tongu, and that constituted the sampling frame. A total of 60 cattle‐only farms, 100 mixed‐flock farms and 10 goat‐only farms were obtained from the study area. However, at the time of sampling, only 40 cattle, 60 mixed‐flock and 5 goat‐only farms were operational and met the inclusion criteria. Farms were stratified according to the number of flock and 3 strata were created, by species and farm as follows: stratum (1) 20 to 100 animals, stratum (2) 101 to 500 animals and stratum (3) 500 and above. Ten animals per species were sampled from stratum 1, 20 from stratum 2 and 50 from stratum 3. Farms were randomly selected within each species of livestock and the total sample size of each category of livestock was proportionally stratified according to the number per flock. On farms where multi‐species livestock were kept, they were considered as a mixed‐flock and sampled as such. Inclusion criteria were livestock aged 6 months and above and a minimum flock size of 20 ruminants. For this study, a farm was considered positive if 10% or more of the sampled animals tested positive or strong positive.

### Variables

Variables measured included demographic characteristics, flock management and contacts with other animals and people, reproductive disorders and health status of the animals. Flock management looked at the system of farming, whether flock was kept extensively, intensively or semi‐intensive. Contact with other animals assessed the presence of other animals on the farms that could have direct access to the housing units (if housed) of the animals. The closeness of human habitation to the housing of the animals was assessed in relation to the distance between the livestock unit and living quarters.

### Sample collection, processing and interpretation

Blood samples were collected aseptically from the jugular vein directly into plain vacuum tubes. Samples were centrifuged at 1500 × *g* for 15 min to obtain sera. Antibodies to *C. burnetii* were detected by a commercial indirect enzyme‐linked immunosorbent assay [ELISA test using microtitre plates pre‐coated with the *C. burnetii* phase I and II strains (IDvet^®^, rue Louis Pasteur, Grabels, France)]. Positive and negative ovine, bovine and caprine control sera were included in each plate. As recommended by the manufacturer, an animal was considered to be ELISA‐strong positive if the optical density (OD) percent was over 80. An OD percent between 50 and 80 was considered positive. A doubtful ELISA result was noted if the OD percent was between 40 and 50, while an OD below or equal to 40 was considered a negative animal. The sensitivity and specificity of the ELISA test kit as provided by the manufacturer (IDvet^®^, rue Louis Pasteur, Grabels, France) was 99% and 98%, respectively.

### Respondents

Farm hands/workers on the selected farms who consented to be interviewed were administered a structured questionnaire which covered risk factors such as proximity of living quarters to farm, frequency of farm visit, occurrence of abortion on farm and disposal of aborted materials. The questionnaire also sought to assess the knowledge of the respondents on Q fever in the area. The inclusion criteria were persons who had worked on the farm for a minimum of 4 weeks and were acquainted with the operation of livestock activities. On each farm, the caretaker and an assistant were invited to participate in the study. Participation in the study was voluntary and no incentives were provided.

### Data collection tools

Field assistants were trained by the Principal Investigator (PI) in the questionnaire administration and basic communication skills. They were drawn from the study region due to their familiarity with the research area. All assistants were veterinary field staff of the Ministry of Food and Agriculture. They were trained on the correct interpretation of each questionnaire in order to ensure consistency of responses and also to reduce interview‐related errors.

### Data analysis

Exploratory data analysis was performed to generate descriptive statistics. Categorical variables were compared using Chi‐square. Student *t*‐test was used to analyse quantitative variables such as the optical density of the sample and binary logistic regression was used to express the relationship between binary dependent variable and independent variables. Data entry and analysis were done using Epi Info version 7.

### Ethical clearance

Permission was sought and obtained from the District Directorate of Agriculture and the Local Livestock Farmers Association. Individual consent was sought from each livestock farmer and participation was voluntary and confidentiality was maintained.

## Results

### Description of farms and livestock

A total of 462 ruminants were sampled from 20 farms and these include 204 cattle, 158 sheep and 100 goats. Out of the 20 farms sampled, 13 (65%) were mixed‐flock, 5 (25%) cattle‐only and 2 (10%) goats‐only. Flock sizes for the various species are displayed in Table 1.

**Table 1:** Flock size and number sampled by species, Tongu area, Ghana

Flock type	Number of farms sampled (%)	Flock size: range (median)	Total animals sampled (%) per species	Number positive (%) per species
Mixed‐flock				
Cattle	5 (25%)	120–900 (150)	144 (70.5%)	30 (20.8%)
Sheep	5 (25%)	45–600 (270)	158 (100%)	45 (28.4%)
Goat	3 (15%)	24–200 (30)	70 (70.0%)	7 (10.0%)
Single‐species				
Cattle	5 (25%)	260–700 (50)	60 (29.5%)	15 (25.0%)
Goat	2 (10%)	30–200 (130)	30 (30.0%)	3 (10.0%)
Total	20 (100%)		462	

The small ruminants (100%) were kept semi‐intensively and were penned during the day and released in the late afternoons for grazing. The cattle (100%) were shepherded for grazing during the mornings and kraaled in the afternoons. Abortions were reported on all the 20 (100%) farms sampled within the last 6 months of sampling. There were no aborted materials seen at the time of sampling.

### Cattle

**Figure 2:**
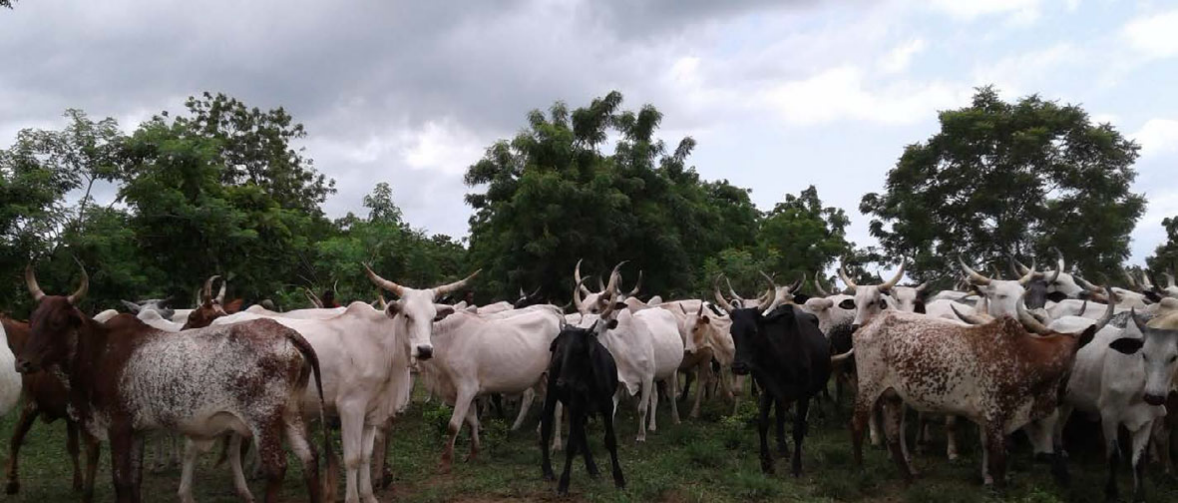
Crosses of breeds between Gudali and Zebu on a cattle farm in the North Tongu district.

Fig. 2 shows the predominant breeds of cattle in the Tongu area and they were Zebu (31; 15%), West African Short Horn (41; 20%), Gudali (20; 10%) and crossbreeds (112; 55%). Breed‐specific prevalence was 24.3% (10/41; *P* = 0.64) for West African Short Horn (WASH), 25.0% (5/20; *P* = 0.53) for Gudali and 26.7% (30/112; *P* = 0.43) for crossbreeds. However, there was no statistical difference between breed of cattle and breed‐specific prevalences. Among the cattle tested, 76% (155/204) were females, out of which 98 (63.2%; *P* = 0.04) were at various stages of lactation. All cattle were apparently healthy except 1 (0.4%) female that had developed inflammation of the carpal and hock joints. Two (0.9%) other cattle presented with dyspnea. Aborted materials from the cattle were disposed within the farm premises on all the cattle farms sampled as reported by the farm hands. Fifty percent of the respondents indicated that aborted materials were buried within the kraals. However, this could not be verified at the time of sampling.

### Sheep and goats

**Figure 3:**
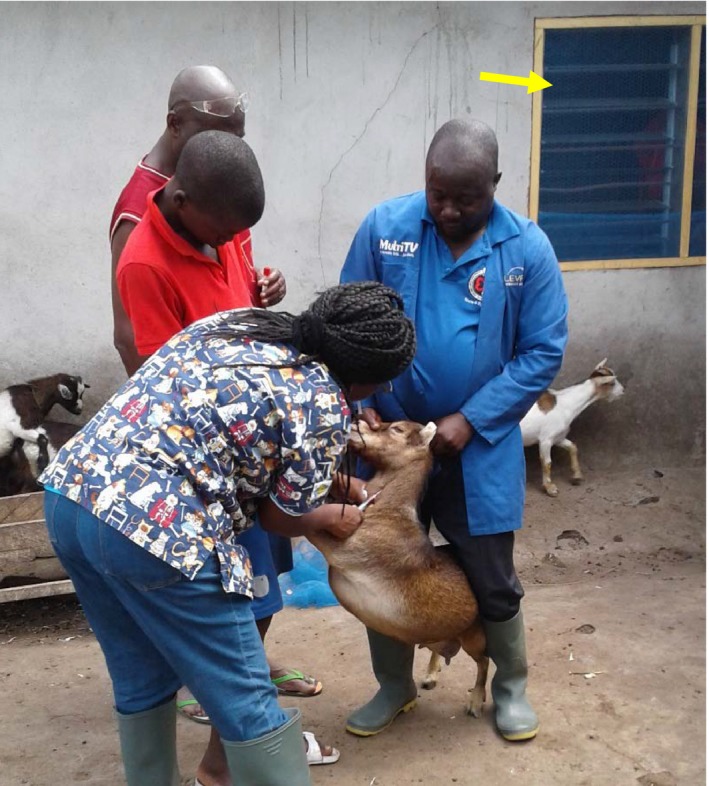
Sheep and goat pen sited next to the window of a bedroom (arrowed yellow) of a farm family.

The sheep and goat breeds included West African Dwarf sheep and goats. It was difficult to ascertain the specific breed of animal sampled due to the mixed breeds that were available. All sheep and goats sampled were deemed healthy. Seventy percent of the sheep and goat farm owners and families lived within 50–100 m from the farms or hired herdsmen lived in close proximity of the farms (Fig. 3). The sheep and goat were mainly kept as backyard livestock farming.

### Prevalence of Q fever

Q fever infection was detected on all farms sampled with an overall prevalence of 21.4% (99/462). Species‐specific prevalence was 28.4% (45/158) for sheep, 22.0% (45/204) for cattle and 10.0% (10/100) for goats.

After adjusting for a sensitivity of 99% and specificity of 98% (IDvet^®^, rue Louis Pasteur, Grabels, France), the prevalence remained unchanged.

**Figure 4:**
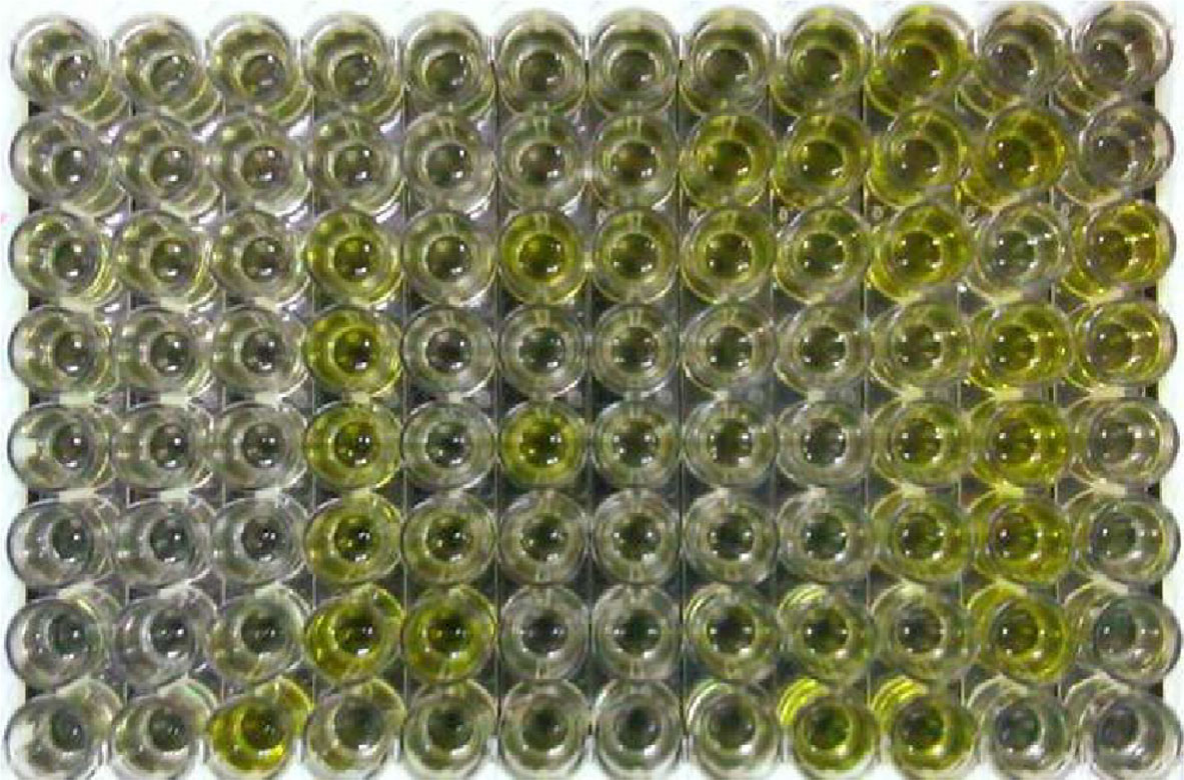
An ELISA plate displaying results from seropositive (yellow) and negative wells in sampled sheep.

Among the species, sheep had the highest species‐specific strong positive of 17.9% and that was statistically significant (*P* = 0.015) as seen in Table 2. The goats had the least of number of positive at 11.0%. High doubtful results (11.3%) were obtained from the cattle and the least seropositive species was goat, with 80% being seronegative (*P* = 0.009). Both sexes, male and female, appeared to have been infected in equal measure irrespective of the number sampled. Fig. 4 shows an ELISA plate from results of seropositive and negative in the tested animals.

**Table 2:** Species‐specific seroprevalence of Q fever in ruminants in the Tongu area

Species	Total sampled/positive	Species‐specific prevalence			
		Strong positive	Positive	Doubtful	Negative
Cattle	204/45; 22.0%	21 (10.3%) *P* = 0.386	24 (11.7%) *P* = 0.275	23 (11.3%) *P* = 0.191	136 (66.7%) *P* = 0.310
Sheep	158/45; 28.4%	27 (17.9%) *P* = 0.015	18 (11.3%) P=0.512	10 (6.33%) P=0.172	103 (65.1%) *P* = 0.287
Goat	100/11; 11.0%	7 (7.0%) *P* = 0.05	4 (4.0%) *P* = 0.023	9 (9.0%) *P* = 0.575	80 (80.0%) *P* = 0.009

### Knowledge on Q fever

A total of 40 participants were invited for the study, 2 per farm and only 2 declined, giving a participation rate of 95%. There were only two permanent veterinary technical officers (non‐DVMs) in charge of the sampled area and they were interviewed making a total of 40 respondents. All respondents were males. Q fever appeared new to the veterinary staff and all farmers interviewed and as result further question could not be posed to the respondents with regards to the level of knowledge on the disease.

### Risk factors

**Figure 5:**
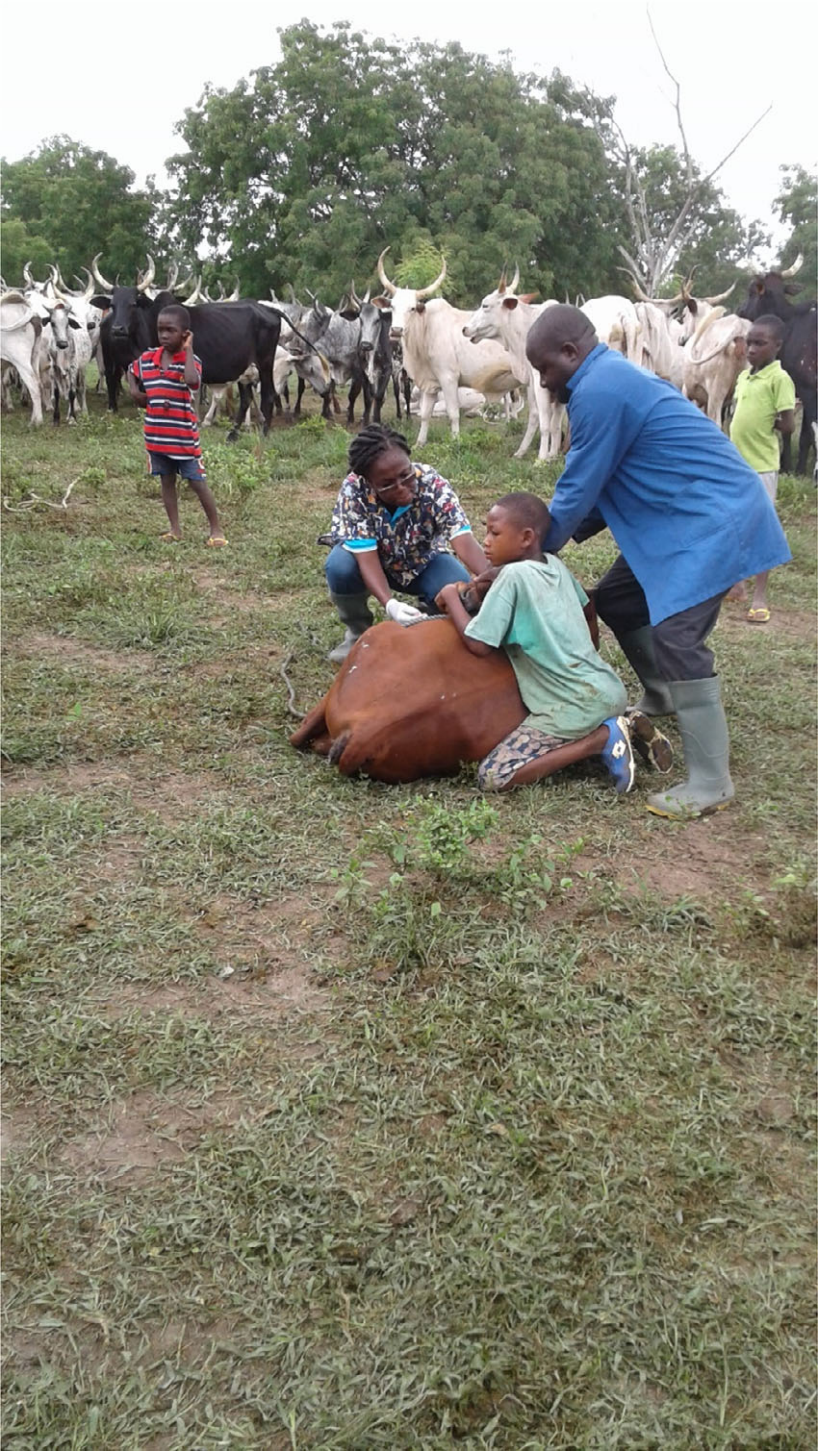
Children assist in the herding of cattle for grazing and other interventions on a farm in North Tongu.

On the cattle farms, children were seen assisting in herding of cattle for grazing (Fig. 5). They were the children of farm owners or hired hands on the farms. The mean (±SD) distance between the kraals of the cattle and living quarters of the farm hands was 129.3 ± 52.2 m. The mean (SD) distance between human settlement and the housing of sheep and goat pens was 63.8 ± 7.9 as exemplified in Fig. 5.

Fetal waste and aborted materials were disposed in the open space in front of the kraals for the cattle and shallow buried for the sheep and goats. Sixty three percent (24/38) of the respondents indicated that they lifted the aborted materials with a plastic bag for disposal. 10.5% (4/38) intimated that abortions occurred when the animals were grazing outside the kraal or pen; hence, the aborted materials were left to rot where they were aborted. The remaining 12 (31.5%; 12/38) indicated that they dug up and buried the fetal materials after abortions.

More than half of the farms kept other animals and these include cats (12; 60%), fowls (15; 75%) and dogs (10; 50%). There was no statistical difference (*P* > 0.05) between farms that kept other livestock and the prevalence of Q fever.

## Discussion

This serological study has shown that Q fever infection was widely spread in sheep, cattle and goat in the Tongu area. An overall prevalence of 21% was very significant given that Q fever has never been reported in the area and in many parts of Ghana. The presence of Q fever infection in all countries neighbouring Ghana, namely Burkina Faso (Ki‐Zerbo *et al*. 2000), Togo (Dean *et al*. 2013) and Cote d'Ivoire (Kanouté *et al*. 2017) was an indication that it could be widespread in Ghana, although not reported and surveilled. According to Tasiame *et al*. (2016), about 80% of the livestock population in the Tongu districts were bred and the area served as a major source of breeding stock to other livestock farms in southern Ghana. The latter together with heavy flow of livestock through transhumance and commercial purposes into northern Ghana suggest that Q fever could be widespread in most livestock communities in Ghana. This calls for the attention and cooperation of the Veterinary and Medical Services in the spirit of “One Health” in order to control it.

Q fever appeared new to the Veterinary staff and farmers in the study area. With the absence of the disease among priority list of diseases under surveillance, it appears not to be in the radar of the Veterinary Authorities and this situation may not be different with the authorities of the Ghana Health Service. This could be a source of worry as an ill farm‐hand with Q fever could easily be misdiagnosed at health facilities in Ghana.

The species‐specific prevalence of 28% in sheep makes this study even more relevant as 70% of the small ruminants were kept as backyard livestock and most farmers lived within less than 50 metres from the tested seropositive ruminants in this study. Given that *C. burnetii* presents asymptomatically in livestock makes it a matter of concern as the infection can spread silently. Tissot‐Dupont *et al*. (2004), found that *C. burtnetii* spread up to 10 miles and the greatest risk of infection was for habitation within 2 km of an outbreak foci. Ratmanov *et al*. (2013) found an association between environmental *C. burtnetii* and human Q fever in Senegal. Given the close proximity of habitation of the farmers to the pens of the livestock, one is left to wonder how much of Q fever infection is contributing to febrile and flu‐like conditions among livestock farmers in the Tongu area?.

The species‐specific prevalences found in this study differed from what was reported in Cote d'Ivoire and Togo. The prevalence for cattle in this study was higher (22%) as compared with 14% in Cote d'Ivoire (Eldin *et al*. 2017; Kanouté *et al*. 2017) and 15% in Togo (Dean *et al*. 2013). However, the prevalence in Ghana was lower than 57% reported in Morocco (Benkirane *et al*. 2015).

In sheep, the prevalence of 28% was higher than reported in Cote d'Ivoire and Togo, however, the prevalence of 10% for goat was comparable to 12% reported in Cote d'Ivoire (Kanouté *et al*. 2017) and 9% in Togo.

The only two published reports on Q fever in Burkina Faso were all in human populations (Gidel & Athawet 1975; Ki‐Zerbo *et al*. 2000). Ki‐Zerbo *et al*. (2000) reported *Coxiella burnetii* in 13% of febrile and hospitalized patients in Burkina Faso and the high incidence was found among 30–60‐year‐olds. However, a search through literature did not yield any livestock prevalence in Burkina Faso. Nonetheless, given that reservoir of Q fever infection is primarily in livestock, it is highly probable that the infection exists in livestock. With free movement of livestock between all three neighbouring countries, the organism *C. burnetii* could be shared freely among these countries.

Abortion was reported to occur on all farms and birth materials were disposed of near the kraals of the animals. Birth materials from the infected animals could serve as a source for infection, not only to the remaining flock but to the farm attendants and inhabitants living in close proximity to the farms. Tasiame *et al*. (2016) reported a prevalence of bovine Brucellosis of 23% in the North Tongu area. How much of the abortions occurring in the study area due to Q fever and/or in concomitant with other abortion causing infections is yet to be determined.

## Conclusion

This study has shown that Q fever infection was prevalent in the Tongu area of the Volta region. Infection was more prevalent in sheep than in cattle and goats. Livestock farmers lived in close proximity to Q fever‐infected livestock and Q fever appeared new to inhabitants of the study area. Awareness needs to be created about the disease in the area and in Ghana as a whole. Further study is required to establish the rate of infection in other parts of the country and access the need for inclusion of Q fever among diseases under surveillance. Collaboration between the Veterinary Services and Ministry of Health is the key to control diseases in the country.

## Source of funding

The USDA – FEP through MSU helped in funding this project and this publication.

## Conflicts of interest

The authors declare no conflicts of interest with regard to the present research.

## Contributions

Conceived and designed study SAMJ and JBK. Field work and sampling SAMJ, KAD and WT. Laboratory analysis IGM, KA and SVS. Wrote the paper SAMJ and JKB. Conceived and designed study SAMJ and JBK. Field work and sampling SAMJ, KAD and WT. Laboratory analysis IGM, KA and SVS. Wrote the paper SAMJ and JKB.

## Ethical statement

This study was conducted following the Institutional guidelines for ethical conduct of research.
